# Hemodiafiltration with endogenous reinfusion for uremic toxin removal in patients undergoing maintenance hemodialysis: a pilot study

**DOI:** 10.1080/0886022X.2024.2338929

**Published:** 2024-04-18

**Authors:** Renhua Lu, Yan Fang, Wangshu Wu, Xiaojun Zeng, Tingting Liu, Yue Qian, Yuanyuan Xie, Yijun Zhou, Leyi Gu

**Affiliations:** Department of Nephrology, Renji Hospital, School of Medicine, Shanghai Jiao Tong University, Shanghai, China

**Keywords:** Maintenance hemodialysis, hemodiafiltration with endogenous reinfusion, uremic toxins, clearance rate, safety

## Abstract

**Objective:**

To delineate the efficacy and safety profile of hemodiafiltration with endogenous reinfusion (HFR) for uremic toxin removal in patients undergoing maintenance hemodialysis (MHD).

**Methods:**

Patients who have been on MHD for a period of at least 3 months were enrolled. Each subject underwent one HFR and one hemodiafiltration (HDF) treatment. Blood samples were collected before and after a single HFR or HDF treatment to test uremic toxin levels and to calculate clearance rate. The primary efficacy endpoint was to compare uremic toxin levels of indoxyl sulfate (IS), λ-free light chains (λFLC), and β_2_-microglobulin (β_2_-MG) before and after HFR treatment. Secondary efficacy endpoints was to compare the levels of urea, interleukin-6 (IL-6), P-cresol, chitinase-3-like protein 1 (YKL-40), leptin (LEP), hippuric acid (HPA), trimethylamine N-oxide (TMAO), asymmetric dimethylarginine (ADMA), tumor necrosis factor-α (TNF-α), fibroblast growth factor 23 (FGF23) before and after HFR treatment. The study also undertook a comparative analysis of uremic toxin clearance between a single HFR and HDF treatment. Meanwhile, the lever of serum albumin and branched-chain amino acids before and after a single HFR or HDF treatment were compared. In terms of safety, the study was meticulous in recording vital signs and the incidence of adverse events throughout its duration.

**Results:**

The study enrolled 20 patients. After a single HFR treatment, levels of IS, λFLC, β_2_-MG, IL-6, P-cresol, YKL-40, LEP, HPA, TMAO, ADMA, TNF-α, and FGF23 significantly decreased (*p* < 0.001 for all). The clearance rates of λFLC, β_2_-MG, IL-6, LEP, and TNF-α were significantly higher in HFR compared to HDF (*p* values: 0.036, 0.042, 0.041, 0.019, and 0.036, respectively). Compared with pre-HFR and post-HFR treatment, levels of serum albumin, valine, and isoleucine showed no significant difference (*p* > 0.05), while post-HDF, levels of serum albumin significantly decreased (*p* = 0.000).

**Conclusion:**

HFR treatment effectively eliminates uremic toxins from the bloodstream of patients undergoing MHD, especially protein-bound toxins and large middle-molecule toxins. Additionally, it retains essential physiological compounds like albumin and branched-chain amino acids, underscoring its commendable safety profile.

## Introduction

End stage renal disease (ESRD) has emerged as a pressing public health challenge of international significance. According to the US Renal Data System (USRDS) 2022 report, by the end of 2020, ESRD cases reached 807,920, representing an upsurge of 1.35-fold compared to figures from 2010 [1]. In 2023, the Chinese National Renal Data System (CNRDS) disclosed that by the end of 2022, 844,265 patients were undergoing maintenance hemodialysis (MHD) in mainland China, highlighting a consistently rising annual trend in patient numbers.

Despite the swift evolution of blood purification technologies and the advent of innovative dialysis membranes, MHD patients persistently contend with an array of challenges. In recent investigations, it has become evident that common uremic toxins, including leptin (LEP) and β_2_-microglobulin (β_2_-MG), exhibit markedly elevated levels in MHD patients. The accumulation of these toxins disrupts the body’s metabolic equilibrium, manifesting in disorders such as malnutrition, amyloidosis, and carpal tunnel syndrome [2]. Moreover, several studies underscore the detrimental influence of a spectrum of uremic toxins on MHD patients. Compounds like indoxyl sulfate (IS), λ-free light chain (λFLC), interleukin-6 (IL-6), P-cresol, chitinase-3-like protein 1 (YKL-40), complement factor D (CFD), hippuric acid (HPA), trimethylamine oxide (TMAO), asymmetric dimethylarginine (ADMA), tumor necrosis factor-α (TNF-α), and fibroblast growth factors 23 (FGF23) are implicated in the suboptimal long-term outcomes of these patients [3–6]. Disconcertingly, current standard blood purification methodologies fall short in effectively eliminating these toxins.

Hemodiafiltration with endogenous reinfusion (HFR) is an innovative therapeutic approach [7] that amalgamates ultrafiltration, diffusion, convection, and adsorption to expeditiously eliminate uremic toxins. These three mechanisms for HFR therapy are grouped in two devices: a dual-chamber and resin adsorbent cartridge. A convective chamber on the top that allows plasma ultrafiltrate to be removed by convection. A diffusive chamber on the bottom, where electrolyte balance is restored, small solutes are removed and overload fluids are ultrafiltrated. The plasma ultrafiltrate, which contains uremic toxins, especially protein-binding toxins, passes into a resin adsorbent cartridge, and changed into a substitution fluid by selective adsorption to return back to the patients. HFR emerges as an optimized, safe, and well-tolerated blood purification strategy, closely mirroring the natural renal filtration process. It not only discriminates in clearing protein-affiliated toxins and middle-to-large molecule toxins but also adeptly sidesteps the hemolytic risks inherent to comprehensive blood adsorption, safeguarding crucial elements like albumin and other beneficial compounds.

Despite its potential, investigations into the application of HFR therapy within Asian cohorts remain scant. A total of 13 studies on HFR in the past 10 years were published [8], and only one of these studies included 37 Chinese subjects [3]. However, this study mainly focused on inflammatory mediators in MHD patients with a single HFR treatment, and did not evaluate the physiological substances, especially the branched-chain amino acids, and the comfort score of HFR treatment. Therefore, we embarked on a prospective, self-controlled study aiming to elucidate the preliminary efficacy of one session of HFR in expunging uremic toxins. Besides, we evaluated the preservation effect of vital physiological agents during the HFR treatment.

## Materials and methods

### Materials

Patients undergoing MHD at the Renji Hospital, School of Medicine, Shanghai Jiao Tong University were enrolled. The inclusion criteria were: (1) aged between 18 and 75 years, with no gender restrictions; (2) undergoing MHD (three times weekly) for a minimum of three months; (3) vascular access should be either through an autologous arteriovenous fistula or a prosthetic graft, with a blood flow rate of at least 200 mL/min; (4) the single pool Kt/V (spKT/V) should be equal to or greater than 1.2; (5) willing to provide informed consent form.

The exclusion criteria were: (1) patients who have been part of other interventional clinical trials in the preceding month; (2) women who are currently pregnant or nursing; (3) those classified as stage 4 by the New York Heart Association (NYHA) or those who have had an acute coronary syndrome or myocardial infarction within the last three months; (4) patients who have experienced active bleeding in the recent two weeks, such as cerebral hemorrhage, gastrointestinal bleeding, or retinal hemorrhage; (5) patients presenting with unstable blood pressure, severe anemia, or at an elevated risk of coagulation; (6) those with severe infections, indicated by tenfold exceeded C-reactive protein levels; (7) a known history of drug addiction or severe mental disorders; and (8) other conditions that might compromise the study determined by investigators.

This study has secured approval from the Ethics Committee of Renji Hospital, School of Medicine, Shanghai Jiao Tong University (LY2023-077-B). All patients provided written informed consent prior to enrollment. This study was registered on the ClinicalTrials.gov under the identifier: NCT06002529.

### Study design and treatment

This was a prospective, self-controlled pilot trial. We employed the Formula Dialysis Therapy machine (Bellco, Italy), to administer the HFR treatment. Integral to the HFR technology are two key components: a dual-chamber filter known as Supra17 (Bellco, Italy) and a resin adsorption column named Suprasorb (Bellco, Italy) (Supplementary Figure 1). For the HDF treatment, the Fresenius 5008S hemodialysis machine and FX1000 hemodiafilter were used.

Each patient received one midweek HFR treatment and one midweek HDF treatment. Midweek treatment means patients who undergoing dialysis on Monday, Wednesday and Friday are treated on Wednesday, and patients who undergoing dialysis on Tuesday, Thursdays and Saturdays are treated on Thursdays. During the study, each subject was treated with HFR first and treated with HDF at least after 2 weeks of HFR treatment. The preferred anticoagulant is low molecular weight heparin. The treatment ensures a blood flow rate that is consistently maintained at or above 200 mL/min. The dialysate flow rate is steadfastly set at 500 mL/min. Standardizing the process, the duration of each hemodialysis session is fixed at 4 h. The ultrafiltration volume is tailored based on each patient’s condition, but it’s advised not to exceed 5% of the patient’s dry weight. Additionally, the filtrate volume is determined automatically, aligning with the current blood flow rate.

### Data collection and assessments

Baseline data of participants were collected, including their age, gender, duration of dialysis, and the causes for MHD. Pertaining to the HFR and HDF treatment, we collected data such as weight before and after treatment, body temperature, blood pressure, heart rate, respiratory rate, treatment duration, blood flow rate, dialysate flow rate, replacement fluid volume, ultrafiltration volume, dosage of low molecular weight heparin, type of vascular access, and incidences of adverse events such as hypotension (systolic pressure <90 mmHg or diastolic pressure <60 mmHg) and coagulation within the dialysis circuitry or the dialyzer.

Further more, blood samples were collected before and after a signal HFR or HDF treatment, and stored in the −80 °C refrigerator. After the study, they were sent to the central laboratory together for testing. Test method is shown as below: Enzyme-Linked Immunosorbent Assay (ELISA) kits were utilized to discern concentrations of several markers, including IS, λFLC, β_2_-MG, urea, IL-6, P-cresol, YKL-40, LEP, HPA, TMAO, ADMA, TNF-α, and FGF23. The uremic toxin clearance rate was calculated using the formula: ([pre-HFR or HDF uremic toxin concentration − post-HFR or HDF uremic toxin concentration]/pre-HFR or HDF uremic toxin concentration) * 100%. In addition to this, both the spKT/V and the urea reduction ratio (URR) [9] were determined. To measure serum albumin levels, albumin diagnostic kits, specifically the BCG method was employed. For gauging concentrations of branched-chain amino acids, valine and isoleucine, high-performance liquid chromatography (HPLC) was used. To ascertain the patient comfort experience during single HFR treatment, the visual analogue scale (VAS) and the fatigue severity scale (FSS) [10] were incorporated (Supplementary Figure 2).

### Endpoints

The primary efficacy endpoint was to compare uremic toxin levels of IS, λFLC, and β_2_-MG before and after HFR treatment, and the clearance of these uremic toxins during a single session of HFR treatment. The secondary efficacy endpoints included assessing the clearance of small molecule toxins such as urea, gauged using spKT/V and URR parameters, and the removal of other significant markers including IL-6, P-cresol, YKL-40, LEP, HPA, TMAO, ADMA, TNF-α, and FGF23. Meanwhile, the comparison of these uremic toxins were performed pre-HFR and post-HFR treatment. And the study also undertook a comparative analysis of uremic toxin clearance between the HFR and HDF treatment. Furthermore, the impact of these treatments on vital physiological substances, specifically serum albumin and branched-chain amino acids, was closely monitored. An important aspect of this study was the assessment of patient comfort during the HFR treatment. In terms of safety, the study was meticulous in recording vital signs and the incidence of adverse events throughout its duration.

### Statistical analysis

Continuous data that follow a normal distribution were expressed as mean ± standard deviation. Comparisons within groups were made using one-way ANOVA or *t*-test. Non-normally distributed continuous data were represented using median and interquartile range (IQR) and compared within groups using the Wilcoxon rank-sum test. Categorical data were represented as frequencies and percentages, and were evaluated using the chi-squared test, Fisher’s exact probability method, and the CMH chi-squared test, as appropriate. Statistical analyses were carried out using the SPSS 20.0 software package (SPSS Inc., Chicago, IL, USA). A *p*-value of <0.05 is considered statistically significant.

## Results

### Baseline characteristics of patients

A total of 20 MHD patients met the inclusion and exclusion criteria, of which 15 were male (75%). The mean age was 48.7 ± 13.6 years, and the mean duration of dialysis was 50.4 ± 43.6 months. The causes for MHD are as follows: 7 cases were due to chronic nephritis (35%, among which 4 had IgA nephropathy and 1 had focal segmental glomerulosclerosis), 4 cases from hypertensive nephrosclerosis (20%), 4 cases from diabetic nephropathy (20%), and in 5 cases the cause remained undetermined (25%).

### Comparison of HFR and HDF treatment information

Throughout the study, each patient underwent one session of HFR and HDF treatment, utilizing low molecular weight heparin for anticoagulation. All treatments were accessed via autologous arteriovenous fistulas. A significant comparison of pretreatment systolic blood pressure showed a notably lower value in HFR than in HDF (*p* = 0.011). Conversely, post-treatment diastolic blood pressure was significantly higher in HFR compared to HDF (*p* = 0.002). Other parameters, including body temperature, blood pressure, heart rate, and respiration, showed no statistical difference pre- and post-treatment in both dialysis modalities (*p* > 0.05) (Table 1). Both HFR and HDF treatments led to a significant decrease in patients’ weight post-treatment compared to pretreatment (*p* < 0.001).

**Table 1.** Comparison of parameters pre- and post-HFR and HDF treatments.

Variables	HFR (*n* = 20)	HDF (*n* = 20)	*p*-Value
Treatment duration (Hours)	4.0	4.0	1.0
Blood flow rate (ml/min)	252.6 ± 17.3	257.9 ± 18.4	0.145
Dialysis fluid flow rate (ml/min)	500	500	1.0
Replacement fluid volume (L)	13.05 ± 1.43	13.26 ± 2.94	0.638
Ultrafiltration volume (L)	2.58 ± 1.0	2.58 ± 0.9	0.991
Low molecular weight heparin dosage (IU)	3968.4 ± 671.7	4084.2 ± 578.6	0.331
Pretreatment weight (Kg)	73.00 ± 13.58	73.11 ± 13.54	0.331
Post-treatment weight (Kg)	70.74 ± 13.56*	70.74 ± 13.55*	1.0
Pretreatment body temperature (°C)	36.16 ± 0.38	36.0 ± 0.0	0.083
Post-treatment body temperature (°C)	36.0 ± 0.00	36.0 ± 0.00	1.0
Pretreatment systolic blood pressure (mmHg)	137.16 ± 24.14	148.79 ± 22.54	0.011
Post-treatment systolic blood pressure (mmHg)	139.21 ± 22.61	143.26 ± 25.37	0.356
Pretreatment diastolic blood pressure (mmHg)	81.95 ± 12.43	77.79 ± 11.51	0.138
Post-treatment diastolic blood pressure (mmHg)	85.05 ± 10.97	77.53 ± 12.24	0.002
Pretreatment heart rate (breaths per minute)	74.32 ± 11.0	75.79 ± 11.7	0.533
Post-treatment heart rate (breaths per minute)	75.95 ± 13.77	73.53 ± 12.51	0.439
Pretreatment respiratory rate (breaths per minute)	18.68 ± 2.56	19.0 ± 2.96	0.695
Post-treatment respiratory rate (breaths per minute)	18.0 ± 1.29	18.58 ± 3.1	0.490

*Compared to pretreatment weight, *p* < 0.001.

### Clearance of IS, λFLC, and β_2_-MG with single HFR treatment

**Figure 1.**
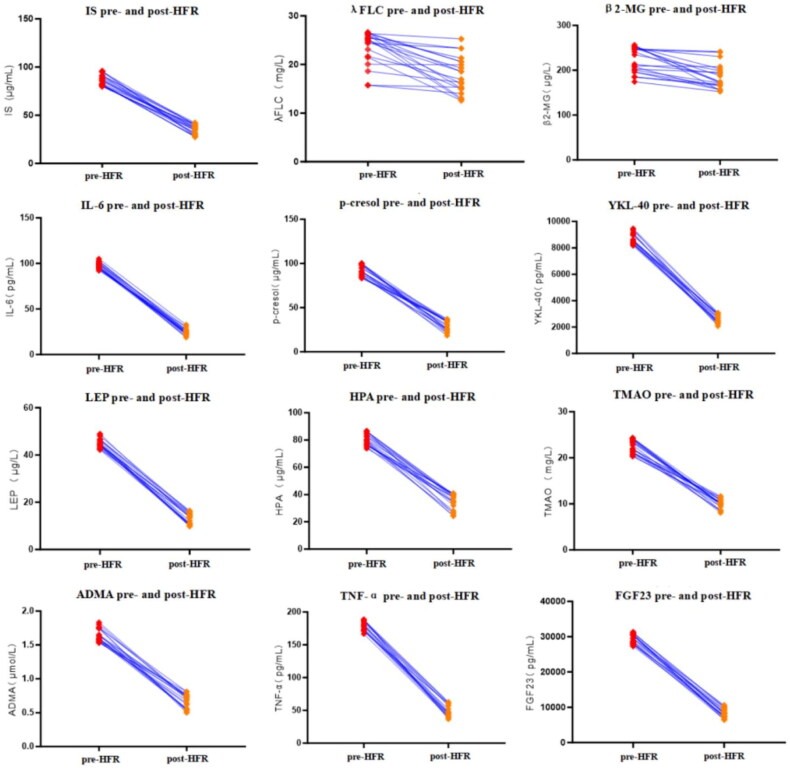
Clearance of IS, λFLC, β_2_-MG, IL-6, P-cresol, YKL-40, LEP, HPA, TMAO, ADMA, TNF-α and FGF23 following HFR treatment.

Upon conducting a singular HFR treatment session, notable reductions were observed post-HFR when compared to pre-HFR measures. Specifically, the levels of IS (μg/ml) manifested a substantial decline from 86.20 ± 5.43 to 34.99 ± 4.94 (*p* < 0.001), marking a 59.3 ± 5.9% clearance rate. Similarly, post-HFR λFLC levels (mg/L) decreased from 23.49 ± 3.48 to 17.90 ± 3.76 (*p* < 0.001), with a 22.9 ± 15.7% reduction. Additionally, β_2_-MG levels (μg/L) following HFR also witnessed a pronounced decline, dropping from 222.58 ± 27.97 to 185.49 ± 27.81 (*p* < 0.001), with a 16.1 ± 11.1% decrease rate (Figure 1).

### Clearance of other uremic toxins with single HFR treatment

Utilizing variables such as spKT/V and URR for evaluating urea clearance post HFR treatment, the findings revealed a mean spKT/*V* value of 1.48 ± 0.37 and a mean URR of 70.87 ± 6.97%. When compared to pre-HFR treatment, post-HFR blood levels of IL-6 (pg/ml) underwent a significant reduction, shifting from 97.61 ± 3.65 down to 24.63 ± 3.61 (*p* < 0.001), with a clearance rate of 74.8 ± 3.5%. Similarly, P-cresol levels in the blood (μg/ml) post-HFR were substantially lower at 24.63 ± 5.97 compared to their baseline count of 91.62 ± 6.07 (*p* < 0.001), indicating a decrease rate of 67.7 ± 7.5%. Lastly, post-HFR concentrations of YKL-40 (pg/ml) also evidenced a marked decrease, transitioning from 8687.31 ± 416.9 to 2619.01 ± 325.0 (*p* < 0.001), which represents a 69.8 ± 3.7% clearance rate (Figure 1).

Following HFR treatment, blood LEP levels (μg/L) showed a substantial reduction, decreasing from 44.99 ± 1.78 to 12.75 ± 2.32 (*p* < 0.001), with a clearance rate of 71.7 ± 4.9%. Likewise, HPA levels in the blood (μg/ml) experienced a significant decline from 80.38 ± 4.29 to 34.62 ± 5.71 (*p* < 0.001), resulting in a clearance rate of 56.9 ± 7.3%. Furthermore, post-HFR blood TMAO concentrations (mg/L) declined notably from 22.51 ± 1.45 to 10.00 ± 1.01 (*p* < 0.001), reflecting a clearance rate of 55.3 ± 6.0% (Figure 1).

In comparison to pre-HFR treatment, the ADMA concentrations in blood (μmol/L) decreased from 1.64 ± 0.09 to 0.66 ± 0.1 (*p* < 0.001), with a clearance rate of 59.6 ± 6.7%. Concurrently, TNF-α levels in the blood (pg/ml) showcased a notable reduction from 178.94 ± 7.20 to 49.37 ± 8.84 (*p* < 0.001), representing a decrease rate of 72.4 ± 4.9%. Additionally, FGF23 blood concentrations (pg/ml) decreased from 29443.0 ± 1271.61 to 8261.5 ± 1276.33 (*p* < 0.001), with a clearance rate of 71.9 ± 4.2% (Figure 1).

### Comparison of uremic toxin clearance between HFR and HDF treatments

In the comparative analysis of uremic toxin clearance, a single session of HFR treatment showed significantly higher clearance rates for λFLC, β_2_-MG, IL-6, LEP, and TNF-α than a single HDF treatment. The respective P-values for these reductions were 0.036, 0.042, 0.041, 0.019, and 0.036 (Table 2).

**Table 2.** Comparison of uremic toxin clearance in HFR and HDF treatments.

Toxin (unit)	Clearance rate in HFR (*n* = 20) (%)	Clearance rate in HDF (*n* = 20) (%)	*p*-Value
IS (μg/ml)	59.3 ± 5.9	53.52 ± 17.1	0.161
λFLC (mg/L)	22.9 ± 15.7	15.18 ± 3.9	0.036
β_2_-MG (μg/L)	16.1 ± 11.1	12.3 ± 3.3	0.042
IL-6 (pg/ml)	74.8 ± 3.5	63.4 ± 19.6	0.041
p-cresol (μg/ml)	67.7 ± 7.5	61.7 ± 15.3	0.152
YKL-40 (pg/ml)	69.8 ± 3.7	64.4 ± 18.2	0.225
LEP (μg/L)	71.7 ± 4.9	62.0 ± 17.1	0.019
HPA (μg/ml)	56.9 ± 7.3	46.0 ± 28.3	0.146
TMAO (mg/L)	55.3 ± 6.0	53.9 ± 16.4	0.700
ADMA (μmol/L)	59.6 ± 6.7	52.8 ± 20.4	0.268
TNF-α (pg/ml)	72.4 ± 4.9	62.5 ± 19.2	0.036
FGF-23 (pg/ml)	71.9 ± 4.2	62.7 ± 21.0	0.111

*Abbreviations*: IS (indoxyl sulfate); λFLC (λ-free light chains); β_2_-MG (β_2_-microglobulin); IL-6 (interleukin-6); YKL-40 (chitinase-3-like protein 1); LEP (leptin); HPA (hippuric acid); TMAO (trimethylamine N-oxide); ADMA (asymmetric dimethylarginine); TNF-α (tumor necrosis factor-α); FGF23 (fibroblast growth factor 23).

### Clearance of serum albumin and branched-chain amino acids in HFR and HDF treatments

For the single HFR treatment, there were no significant differences in the levels of serum albumin (40.75 ± 5.50g/L vs. 41.30 ± 7.05g/L, *p* = 0.785), valine (mg/L) [8551.0 (6436.75, 9213.75) vs. 1569.0 (1078.0, 9333.0, *p* = 0.121)], and isoleucine (mg/L) [2322.5 (1663.0, 3108.75) vs. 3165.0 (2566.0, 4055.0), *p* = 0.121] before and after the treatment (Table 3).

**Table 3.** Clearance of serum albumin and branched-chain amino acids in HFR and HDF treatments.

Variables	Pre-HFR (*n* = 20)	Post-HFR (*n* = 20)	Pre-HDF (*n* = 20)	Post-HDF (*n* = 20)
Serum albumin (g/L)	40.75 ± 5.50	41.30 ± 7.05	48.43 ± 6.16	35.89 ± 6.3*
Valine (mg/L)	8551.0 (6436.75, 9213.75)	1569.0 (1078.0, 9333.0)	6634.5 (1744.75, 9398.0)	1214.5 (1073.25, 7222.75)
Isoleucine (mg/L)	2322.5 (1663.0, 3108.75)	3165.0 (2566.0, 4055.0)	3670.5 (1298.5, 7005.25)	3168.5 (1440.5, 6265.0)

*Compared to pre-HDF, *p* = 0.000.

Conversely, following single HDF treatment, there was a significant decrease in serum albumin levels (48.43 ± 6.16g/L vs. 35.89 ± 6.3g/L, *p* = 0.000). However, the levels of valine (mg/L) [6634.5 (1744.75, 9398.0) vs. 1214.5 (1073.25, 7222.75), *p* = 0.051)] and isoleucine (mg/L) [3670.5 (1298.5, 7005.25) vs. 3168.5 (1440.5, 6265.0), *p* = 0.404)] showed no significant differences before and after the treatment (Table 3).

### Assessment of comfort during HFR treatment

The comfort experience during HFR treatment was evaluated using both the VAS and the FSS. The recorded mean VAS comfort score was 7.4 ± 1.54 points. The mean FSS score was 35.95 ± 16.37 points, with 11 (55%) participants scoring below 36.

### Vital signs and adverse events throughout the study

Throughout the study, the patients showcased consistent vital signs, such as body temperature, blood pressure, heart rate, and respiration rates, pre- and post-HFR and HDF treatment (*p* > 0.05) (Table 1). Furthermore, during the HFR treatment, adverse events, including hypotension, were absent among the patients. Notably, post-HFR examinations revealed no coagulation within the dialysis circuitry or the dialyzer.

## Discussion

Although the rapid development of dialysis technology including the improving of dialysis membranes and the increasing of removal uremic toxins, the quality of life and survival of the MHD population remains low [11]. Inadequate clearance of uremic toxins, with a particular emphasis on protein-bound and middle-molecule toxins (25–50KDa), may be one of the main reasons. Furthermore, existing literature underscores the formidable challenge of efficaciously eliminating these toxins [12–16]. This necessitates the exploration and integration of innovative blood purification techniques adept at efficaciously purging these toxins.

The combination of diffusion, convection and adsorption of HFR in a single therapeutic scheme may benefit the removal of these toxins. In our study, we identified several protein-bound small-molecule uremic toxins, including IS, P-cresol, HPA, TMAO, and ADMA. Current literature have highlighted that an accumulation of these toxins is implicated in the progression of peripheral arterial diseases and neurological complications, accelerated the onset of atherosclerosis and intensify cardiovascular threats, increased rates of hospitalization and a surge in mortality [5,17–20]. Unfortunately, the conjugation of these toxins with plasma proteins limits their eradication through conventional hemodialysis. Our data indicate that following one session HFR treatment, there was a significant reduction in the levels of IS, P-cresol, HPA, TMAO, and ADMA, with decrement rates being 59.3 ± 5.9%, 67.7 ± 7.5%, 56.9 ± 7.3%, 55.3 ± 6.0%, and 59.6 ± 6.7%, respectively. These results suggest that HFR treatment may remove protein-bound small-molecule uremic toxins through convection and adsorption. Our observation is consistent with existing literature [3], positing that HFR, by leveraging its resin adsorption column, can adeptly remove protein-bound small-molecule uremic toxins present in the filtrate.

In the present study, we explored the role of large middle-molecule toxins, including λFLC, IL-6, TNF-α, YKL-40, and FGF23. Current literature underscores that an excessive buildup of these toxins has implications, leading to persistent inflammation and pronounced vascular calcification, linked to an augmented risk of cardiovascular incidents and an intensified mortality in MHD subjects [21–28]. While these large middle-molecule toxins can be effectively filtered using advanced high-flux or high cutoff membrane technologies, the conundrum of preserving essential physiological entities like albumin and branched-chain amino acids persists [29]. This study discovered that under similar treatment dosages (replacement fluid volume + ultrafiltration volume), a single HFR treatment demonstrated significantly superior clearance rates in λFLC, IL-6, and TNF-α compared to a single HDF treatment. This result suggests that in addition to convection, adsorption plays a key role to regeneration of plasma ultrafiltrate using an adsorbing resin allows effective removal of large middle-molecule toxins in HFR treatment. Remarkably, levels of serum albumin and branched-chain amino acids remained unchanged post-HFR treatment when compared to pretreatment levels. These findings suggest that HFR, through its resin adsorption column, is potentially more effective than HDF in adsorbing large middle-molecule uremic toxins from the filtrate, while concurrently preserving vital physiological substances like albumin. However, this hypothesis requires further validation through prospective, large-scale clinical studies with extended follow-up periods.

In this investigation, we examined small middle-molecule toxins, specifically β_2_-MG and LEP. For patients undergoing MHD, the detrimental consequences of these toxins accumulation encompass dialysis-associated amyloidosis, augmented cardiovascular disease risk, chronic inflammatory conditions, neoplastic disorders, and protein-energy malnutrition [30–32]. Our study indicates that, with similar treatment dosages (replacement fluid volume + ultrafiltration volume), the clearance rates of β_2_-MG and LEP following a single HFR treatment were significantly superior to those in a single HDF treatment. This finding highlights the potential of HFR treatment to effectively clear small middle-molecule uremic toxins, potentially surpassing the efficacy of HDF treatment in this regard. It merits attention that our study documented a decline rate for β_2_-MG, which is less pronounced than the 43.6% delineated in previous literature [3]. This variance may be due to some of our patients having undergone a relatively brief span of dialysis, culminating in diminished pre-dialysis toxin concentrations. Such observations warrant corroborative studies for comprehensive validation.

spKT/V and URR serve as robust indicators for gauging the elimination of water-soluble, small-molecule toxins, notably urea. Research has identified an spKT/*V* < 1.2 or URR < 65% as a harbinger of adverse outcomes in MHD patients [9,33]. Our research demonstrates that post-HFR treatment, the mean spKT/V was 1.48 ± 0.37, and the mean URR was 70.87 ± 6.97%. These results underscore the efficacy of HFR in expeditiously clearing water-soluble, small-molecule uremic toxins, leveraging the diffusion dynamics in the lower chamber.

Previous explorations into the sufficiency of blood purification predominantly centered on the purgation of uremic toxins, with scant attention to the comfort experienced during dialysis. In a recent shift, the Kidney Disease Improving Global Outcomes (KDIGO) entity advocated for a comprehensive management approach addressing the symptomatic burdens associated with MHD, encapsulating the element of dialytic comfort [34]. Previous inquiries have corroborated that compromised comfort during dialysis, especially manifesting as fatigue, bears significant correlations with cardiovascular ailments, elevated mortality, depressive moods, and a diminished quality of life [35]. Our investigation ascertained a comfort VAS score of 7.4 ± 1.54, with a noteworthy 55% of participants recording an FSS score below 36, underscoring the superior comfort accompanying HFR sessions. Parallelly, the stability of vital signs throughout the study’s trajectory and the absence of adverse event accentuates the impressive safety profile of HFR therapy.

This study has several limitations. Firstly, our investigation was conducted at a single center and involved a modest sample size, potentially impinging upon the broader applicability of our conclusions. Secondly, our examination centered only on toxin clearance immediately before and after a solitary HFR session, and it does not furnish insights into the extended clearance rates of protein-bound toxins and large middle-molecule toxins through repeat HFR interventions.

In conclusion, our preliminary findings posit that HFR therapy adeptly eliminates uremic toxins from the bloodstream of patients on MHD, notably targeting protein-bound toxins and large middle-molecule toxins. It concurrently conserves vital physiological entities, such as albumin and branched-chain amino acids, underscoring its commendable safety profile.

## Supplementary Material

Supplemental Material

## Data Availability

Data are not publicly available due to ethical reasons. Further inquiries can be directed to the corresponding author.

## References

[1] 2022 USRDS annual data report; 2022. http://usrds-adr.niddk.nih.gov/2022/end-stage-renal-disease/1-incidence-prevalence-patient-characteristics-and-treatment-modalities.

[2] Kim S, Oh KH, Chin HJ, et al. Effective removal of leptin via hemodiafiltration with on-line endogenous reinfusion therapy. Clin Nephrol. 2009;72(6):1–9. doi: 10.5414/cnp72442. 19954721

[3] Chen X, Shen B, Cao X, et al. Acute effect of one session of hemodiafiltration with endogenous reinfusion on uremic toxins and inflammatory mediators. Int J Artif Organs. 2020;43(7):437–443. doi: 10.1177/0391398819899102. 31942823

[4] Donati G, Angeletti A, Cappuccilli M, et al. Efficacy of supra-HFR in removing FGF23 and cytokines: a single session analysis. In Vivo. 2022;36(4):1769–1776. doi: 10.21873/invivo.12890. 35738602 PMC9301447

[5] Lim YJ, Sidor NA, Tonial NC, et al. Uremic toxins in the progression of chronic kidney disease and cardiovascular disease: mechanisms and therapeutic targets. Toxins. 2021;13(2):142. doi: 10.3390/toxins13020142. 33668632 PMC7917723

[6] Murgia S, Quattrocchio G, Forneris G, et al. Management of acute kidney injury in frail patients with biopsy-proven cast nephropathy: a combined approach with chemotherapy plus supra-hemodiafiltration with post-adsorption endogenous reinfusion. J Nephrol. 2022;35(4):1243–1249. doi: 10.1007/s40620-021-01226-4. 34982413

[7] Lou Wratten M, Ghezzi PM. Hemodiafiltration with endogenous reinfusion. Contrib Nephrol. 2007;158:94–102. doi: 10.1159/000107239. 17684347

[8] Molina P, Goicoechea M, Huarte E, et al. Hemodiafiltration with endogenous reinfusion of the regenerated ultrafiltrate (HFR): towards a convective, diffusive, and adsorptive dialysis. Nefrologia. 2023;43(6):688–702. doi: 10.1016/j.nefroe.2023.12.003. 38176980

[9] National Kidney Foundation. KDOQI clinical practice guideline for hemodialysis adequacy: 2015 update. Am J Kidney Dis. 2015;66(5):884–930. doi: 10.1053/j.ajkd.2015.07.015. 26498416

[10] Bonner A, Wellard S, Caltabiano M. The impact of fatigue on daily activity in people with chronic kidney disease. J Clin Nurs. 2010;19(21–22):3006–3015. doi: 10.1111/j.1365-2702.2010.03381.x. 21040007

[11] Blankestijn PJ, Vernooij RWM, Hockham C, et al. Effect of hemodiafiltration or hemodialysis on mortality in kidney failure. N Engl J Med. 2023;389(8):700–709. doi: 10.1056/NEJMoa2304820. 37326323

[12] Fujii H, Goto S, Fukagawa M. Role of uremic toxins for kidney, cardiovascular, and bone dysfunction. Toxins. 2018;10(5):202. doi: 10.3390/toxins10050202. 29772660 PMC5983258

[13] Yamamoto S. Molecular mechanisms underlying uremic toxin-related systemic disorders in chronic kidney disease: focused on β2-microglobulin-related amyloidosis and indoxyl sulfate-induced atherosclerosis-Oshima award address 2016. Clin Exp Nephrol. 2019;23(2):151–157. doi: 10.1007/s10157-018-1588-9. 29869756 PMC6510801

[14] Liabeuf S, Cheddani L, Massy ZA. Uremic toxins and clinical outcomes: the impact of kidney transplantation. Toxins. 2018;10(6):229. doi: 10.3390/toxins10060229. 29874852 PMC6024850

[15] Wolley M, Jardine M, Hutchison CA. Exploring the clinical relevance of providing increased removal of large middle molecules. Clin J Am Soc Nephrol. 2018;13(5):805–814. doi: 10.2215/CJN.10110917. 29507008 PMC5969479

[16] Yamamoto S, Kazama JJ, Wakamatsu T, et al. Removal of uremic toxins by renal replacement therapies: a review of current progress and future perspectives. Ren Replace Ther. 2016;2(1):43. doi: 10.1186/s41100-016-0056-9.

[17] Leong SC, Sirich TL. Indoxyl sulfate-review of toxicity and therapeutic strategies. Toxins. 2016;8(12):358. doi: 10.3390/toxins8120358. 27916890 PMC5198552

[18] Lin T-Y, Chou H-H, Huang H-L, et al. Indoxyl sulfate and incident peripheral artery disease in hemodialysis patients. Toxins. 2020;12(11):696. doi: 10.3390/toxins12110696. 33147880 PMC7693838

[19] Bammens B, Evenepoel P, Keuleers H, et al. Free serum concentrations of the protein-bound retention solute p-cresol predict mortality in hemodialysis patients. Kidney Int. 2006;69(6):1081–1087. doi: 10.1038/sj.ki.5000115. 16421516

[20] Spence JD, Urquhart BL. Cerebrovascular disease, cardiovascular disease, and chronic kidney disease: interplays and influences. Curr Neurol Neurosci Rep. 2022;22(11):757–766. doi: 10.1007/s11910-022-01230-6. 36181576

[21] Bourguignon C, Chenine L, Bargnoux AS, et al. Hemodiafiltration improves free light chain removal and normalizesκ/λratio in hemodialysis patients. J Nephrol. 2016;29(2):251–257. doi: 10.1007/s40620-015-0207-z. 26022721

[22] Esparvarinha M, Nickho H, Mohammadi H, et al. The role of free kappa and lambda light chains in the pathogenesis and treatment of inflammatory diseases. Biomed Pharmacother. 2017;91:632–644. doi: 10.1016/j.biopha.2017.04.121. 28494417

[23] Libby P. Targeting inflammatory pathways in cardiovascular disease: the inflammasome, interleukin-1, interleukin-6 and beyond. Cells. 2021;10(4):951. doi: 10.3390/cells10040951. 33924019 PMC8073599

[24] Chen Z, Wang Y. Interleukin-6 levels can be used to estimate cardiovascular and all-cause mortality risk in dialysis patients: a meta-analysis and a systematic review. Immun Inflamm Dis. 2023;11(4):e818. 37102647 10.1002/iid3.818 PMC10132186

[25] Castillo-Rodríguez E, Pizarro-Sánchez S, Sanz AB, et al. Inflammatory cytokines as uremic toxins: “Ni son todos los que estan, ni estan todos los que son”. Toxins. 2017;9(4):114. doi: 10.3390/toxins9040114. 28333114 PMC5408188

[26] Vega A, Sanchez-Niño MD, Ortiz A, et al. The new marker YKL-40, a molecule related to inflammation, is associated with cardiovascular events in stable haemodialysis patients. Clin Kidney J. 2019;13(2):172–178. doi: 10.1093/ckj/sfz056. 32296521 PMC7147298

[27] Zeng D, Zha A, Lei Y, et al. Correlation of serum FGF23 and chronic kidney disease-mineral and bone abnormality markers with cardiac structure changes in maintenance hemodialysis patients. Evid Based Complement Alternat Med. 2023;2023:6243771–6243777. doi: 10.1155/2023/6243771. 37089720 PMC10118877

[28] Ketteler M, Block GA, Evenepoel P, et al. Executive summary of the 2017 KDIGO chronic kidney disease-mineral and bone disorder (CKD-MBD) guideline update: what’s changed and why it matters. Kidney Int. 2017;92(1):26–36. doi: 10.1016/j.kint.2017.04.006. 28646995

[29] El-Sayed H, Abdelmohsen W, Abdelmegied S, et al. High-Flux dialyzer 2.6 m2 is promising for free light chains removal in high-flux hemodialysis and in hemodiafiltration. EJI. 2022;29(4):106–114. doi: 10.55133/eji.290410. 36206153

[30] Argyropoulos CP, Chen SS, Ng Y-H, et al. Rediscovering beta-2 microglobulin as a biomarker across the spectrum of kidney diseases. Front Med. 2017;4:73. doi: 10.3389/fmed.2017.00073. PMC5471312 28664159

[31] Cordeiro ISF, Cordeiro L, Wagner CS, et al. High-flux versus high retention-onset membranes: *in vivo* small and middle molecules kinetics in convective dialysis modalities. Blood Purif. 2020;49(1–2):8–15. doi: 10.1159/000502082. 31362299

[32] Teta D. Adipokines as uremic toxins. J Ren Nutr. 2012;22(1):81–85. doi: 10.1053/j.jrn.2011.10.029. 22200420

[33] Daugirdas JT. Kt/V (and especially its modifications) remains a useful measure of hemodialysis dose. Kidney Int. 2015;88(3):466–473. doi: 10.1038/ki.2015.204. 26176827

[34] Mehrotra R, Davison SN, Farrington K, et al. Managing the symptom burden associated with maintenance dialysis: conclusions from a kidney disease: improving global outcomes (KDIGO) controversies conference. Kidney Int. 2023;104(3):441–454. doi: 10.1016/j.kint.2023.05.019. 37290600

[35] van Sandwijk MS, Al Arashi D, van de Hare FM, et al. Fatigue, anxiety, depression and quality of life in kidney transplant recipients, haemodialysis patients, patients with a haematological malignancy and healthy controls. Nephrol Dial Transplant. 2019;34(5):833–838. doi: 10.1093/ndt/gfy103. 29726909

