# Supplementary material for: Hemodiafiltration with endogenous reinfusion for uremic toxin removal in patients undergoing maintenance hemodialysis: a pilot study
Source: Ren Fail. 2024 Apr 18;46(1):2338929. doi: 10.1080/0886022X.2024.2338929 (PMC11028005; doi:10.1080/0886022X.2024.2338929)
Supplement: Supplemental Material [file IRNF_A_2338929_SM3041.pdf]

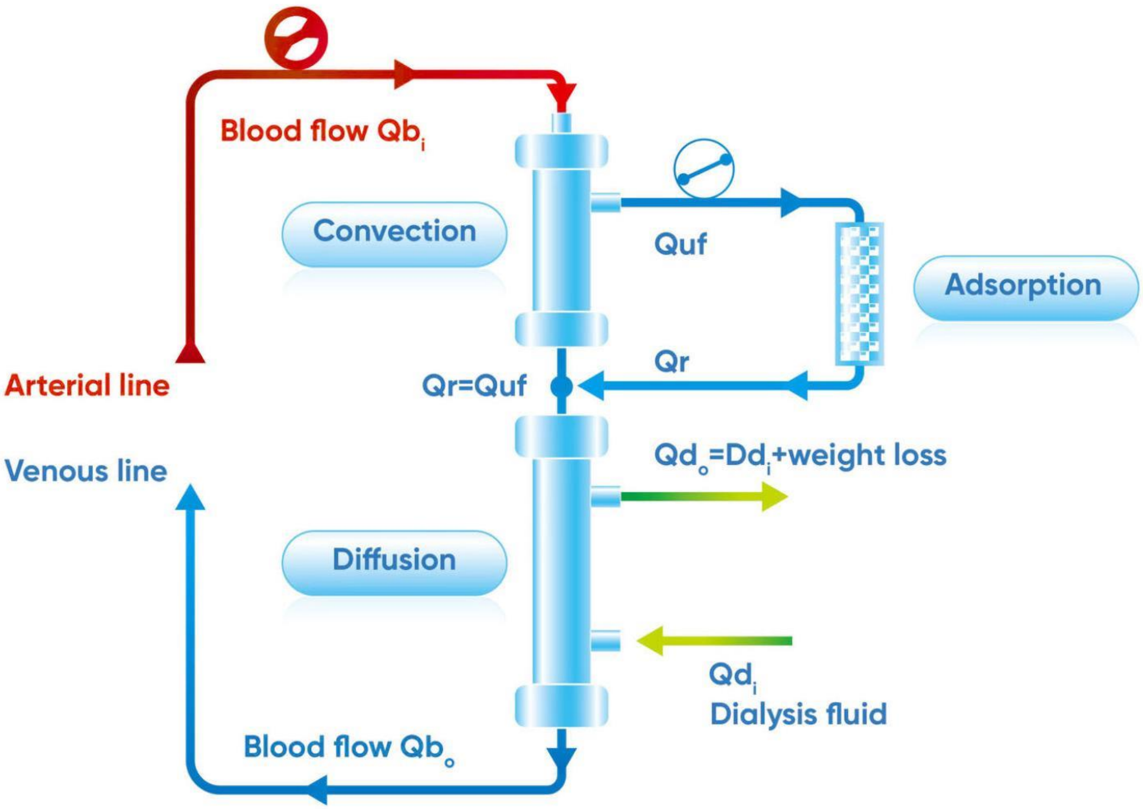
Blood flow Qb$_i$
Convection
Quf
Adsorption
Qr
Arterial line
Qr=Quf
Qd$_o$=Dd$_i$+weight loss
Venous line
Diffusion
Qd$_i$
Dialysis fluid
Blood flow Qb$_o$

1、VAS for comfort

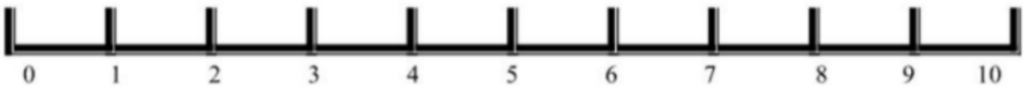

Least comfortable Most comfortable

2、FSS

Nine items	Agreement level (Score)
①My motivation is lower when I am fatigued.	
②Exercise brings on my fatigue.	
③I am easily fatigued.	
④Fatigue interferes with my physical functioning.	
⑤Fatigue causes frequent problems for me.	
⑥My fatigue prevents sustained physical functioning.	
⑦Fatigue interferes with carrying out certain duties and responsibilities.	
⑧Fatigue is among my most disabling symptoms.	
⑨Fatigue interferes with my work, family, or social life.	
Total score	
Average score	

1	2	3	4	5	6	7
Strongly disagree						Strongly agree

A score of ≥36 indicates the presence of fatigue; the higher the score, the more severe the fatigue.
